# Tumor motion changes in stereotactic body radiotherapy for liver tumors: an evaluation based on four-dimensional cone-beam computed tomography and fiducial markers

**DOI:** 10.1186/s13014-017-0799-7

**Published:** 2017-03-23

**Authors:** Yoshinobu Shimohigashi, Ryo Toya, Tetsuo Saito, Osamu Ikeda, Masato Maruyama, Keisuke Yonemura, Yuji Nakaguchi, Yudai Kai, Yasuyuki Yamashita, Natsuo Oya, Fujio Araki

**Affiliations:** 1 Department of Radiological Technology, Kumamoto University Hospital, 1-1-1 Honjo, Chuo-ku, Kumamoto, 860-8556 Japan; 2 Graduate School of Health Sciences, Kumamoto University, 4-24-1 Kuhonji, Chuo-ku, Kumamoto, 862-0976 Japan; 3 Department of Radiation Oncology, Kumamoto University Hospital, Kumamoto, Japan; 4 Department of Diagnostic Radiology, Kumamoto University Hospital, Kumamoto, Japan; 5 Department of Health Sciences, Faculty of Life Sciences, Kumamoto University, Kumamoto, Japan

**Keywords:** Liver tumor, Four-dimensional cone-beam computed tomography, Image-guided radiotherapy, Internal target volume, Stereotactic body radiotherapy

## Abstract

**Background:**

For stereotactic body radiation therapy (SBRT) of liver tumors, tumor motion induced by respiration must be taken into account in planning and treatment. We evaluated whether liver tumor motion at the planning simulation represents liver tumor motion during SBRT, and estimated inter- and intrafractional tumor motion changes in patients undergoing liver SBRT.

**Methods:**

Ten patients underwent four-dimensional cone-beam computed tomography (4D-CBCT) image-guided liver SBRT with abdominal compression (AC) and fiducial markers. 4D-CBCT was performed to evaluate liver tumor motion at the planning simulation, pre-, and post-SBRT. The translational distances at the center position of the fiducial markers from all 10 phases on the 4D-CBCT images were measured as the extent of the liver tumor motion in the left-right (LR), anterior-posterior (AP), and superior-inferior (SI) directions. Pearson correlation coefficients were calculated to evaluate the correlation between liver tumor motion of the planning simulation and the mean liver tumor motion of the pre-SBRT. Inter- and intrafractional liver tumor motion changes were measured based on the 4D-CBCT of planning simulation, pre-, and post-SBRT. Significant inter- and intrafractional changes in liver tumor motion were defined as a change of >3 mm.

**Results:**

The mean (± SD) liver tumor motion of the planning simulation 4D-CBCT was 1.7 ± 0.8 mm, 2.4 ± 2.2 mm, and 5.3 ± 3.3 mm, in the LR, AP, and SI directions, respectively. Those of the pre-SBRT 4D-CBCT were 1.2 ± 0.7 mm, 2.3 ± 2.3 mm, and 4.5 ± 3.8 mm, in the LR, AP, and SI directions, respectively. There was a strong significant correlation between liver tumor motion of the planning simulation and pre-SBRT in the LR (*R* = 0.7, *P* < 0.01), AP (*R* = 0.9, *P* < 0.01), and SI (*R* = 0.9, *P* < 0.01) directions. Significant inter- and intrafractional liver tumor motion changes occurred in 10 and 2% of treatment fractions, respectively.

**Conclusions:**

Liver tumor motion at the planning simulation represents liver tumor motion during SBRT. Inter- and intrafractional liver tumor motion changes were small in patients with AC.

## Background

Stereotactic body radiation therapy (SBRT) for liver tumors has been introduced as alternatives to standard treatment modalities such as surgical resection and radiofrequency ablation. SBRT delivers a highly conformal, potent dose of radiation to the tumor in a limited number of fractions while minimizing radiation damage to organs at risk [1]. To prescribe an accurate radiation dose to liver tumors, inter- and intrafractional motion, induced by respiration, must be taken into account during planning simulation and treatment [2–5]. However, unlike lung tumors, liver tumors are difficult to visualize due to the lack of soft tissue contrast of image-guided radiotherapy modalities, such as computed tomography (CT) and cone-beam CT (CBCT) [6–8]. To address this issue, metal fiducial markers, which are implanted in or near the tumor, are used as tumor surrogates. Image guidance with fiducial markers is reported as a more accurate method than image guidance with the liver contour or diaphragm position [2, 8–11].

The value of four-dimensional computed tomography (4D-CT) is reported to define tumor contours and the internal target volume (ITV) during respiratory movement in the RT treatment planning, assuming that the tumor motion of 4D-CT at the planning session represents the tumor motion throughout the course of SBRT [3–5, 12–14]. However, 4D-CT is not installed in all institutions. As a system for performing respiration-corrected CBCT, 4D-CBCT has been used for image-guided SBRT for lung and liver tumors [4, 15–18]. Park et al. [18] have performed 4D-CBCT using motion tracking of fiducial markers during liver SBRT. They reported that 4D-CBCT could significantly reduce motion-induced blurring of fiducial markers and liver anatomy, and could be useful for image-guided liver SBRT. Using 4D-CBCT, Case et al. [4] reported that inter- and intrafractional liver motion changes during liver SBRT in 29 patients were <3 mm in 80% of the fractions. However, they evaluated liver motion changes based on the diaphragm position, which may be inappropriate for the evaluation of liver tumor motion.

In the previous report, intrafractional tumor motion during free-breathing was investigated using a real-time tumor tracking radiotherapy (RTRT) system and fiducial markers for liver SBRT [2], but changes in the tumor motion throughout the course of treatment were not been fully evaluated. In this study, we evaluated whether liver tumor motion at the planning simulation represents liver tumor motion during SBRT, and estimated changes of inter- and intrafractional tumor motion in patients undergoing liver SBRT with abdominal compression (AC) using 4D-CBCT and fiducial markers.

## Methods

### Patients

This retrospective study was approved by the institutional review board of our hospital. Prior informed consent for treatment and the use of 4D-CBCT and its images for the study was obtained from all patients. Between May 2014 and May 2016, 11 consecutive patients underwent 4D-CBCT image-guided liver SBRT. Of these, 10 patients were included in this study. One patient was excluded because she refused AC because it caused discomfort. Of the 10 patients, 5 patients had hepatocellular carcinoma, 2 had cholangiocellular carcinoma, and 3 had liver metastases. Liver tumors were located in the segment 4 in 2, 5 in 2, 6 in 1, 7 in 2, and 8 in 3 patients. The mean value of gross tumor volume (GTV) was 10.9 mL (range, 0.4–45.9 mL). All patients had 1–2 gold fiducial markers 2 mm in diameter (iGold; Medikit, Tokyo, Japan) implanted in the liver using the percutaneous transhepatic approach [19].

### Planning simulation CT

All patients lay supine with their arms raised and were immobilized with a system consisting of a vacuum bag, thermoplastic body shell, and carbon base plate with a size of 185 × 50 × 3 cm (length × width × height) (ESN-1800; Engineering System, Nagano, Japan). Patients were instructed to breathe shallowly for 1 minute to form a body shell. AC was applied in all patients to achieve reproducible tumor motion for planning and treatment; an in-house beaded cushion was inserted inside the formed body shell for AC. Multiphasic contrast-enhanced CT and slow CT were performed during the free-breathing using a LightSpeed RT CT scanner (GE Medical Systems, Waukesha, WI). Contrast-enhanced scans with the helical mode (rotation time: 1 s) were performed 30, 45, 70, and 180 s after the intravenous administration of a contrast agent (Iopamiron 300, Bayer Schering Pharma, Osaka, Japan) at a rate of 3 mL/s. A slow CT scan with the axial mode (rotation time: 4 s) was performed immediately after the completion of these contrast-enhanced scans. Both sets of CT data were reconstructed in a field of view (FOV) of 65 cm with a 2.5-mm slice thickness. All CT images were exported to the Pinnacle^3^ treatment planning system (version 9.2; Phillips Radiation Oncology Systems, Fitchburg, WI) and were registered by hardware arrangement.

### Estimation of the tumor motion on 4D-CBCT

**Fig. 1:**
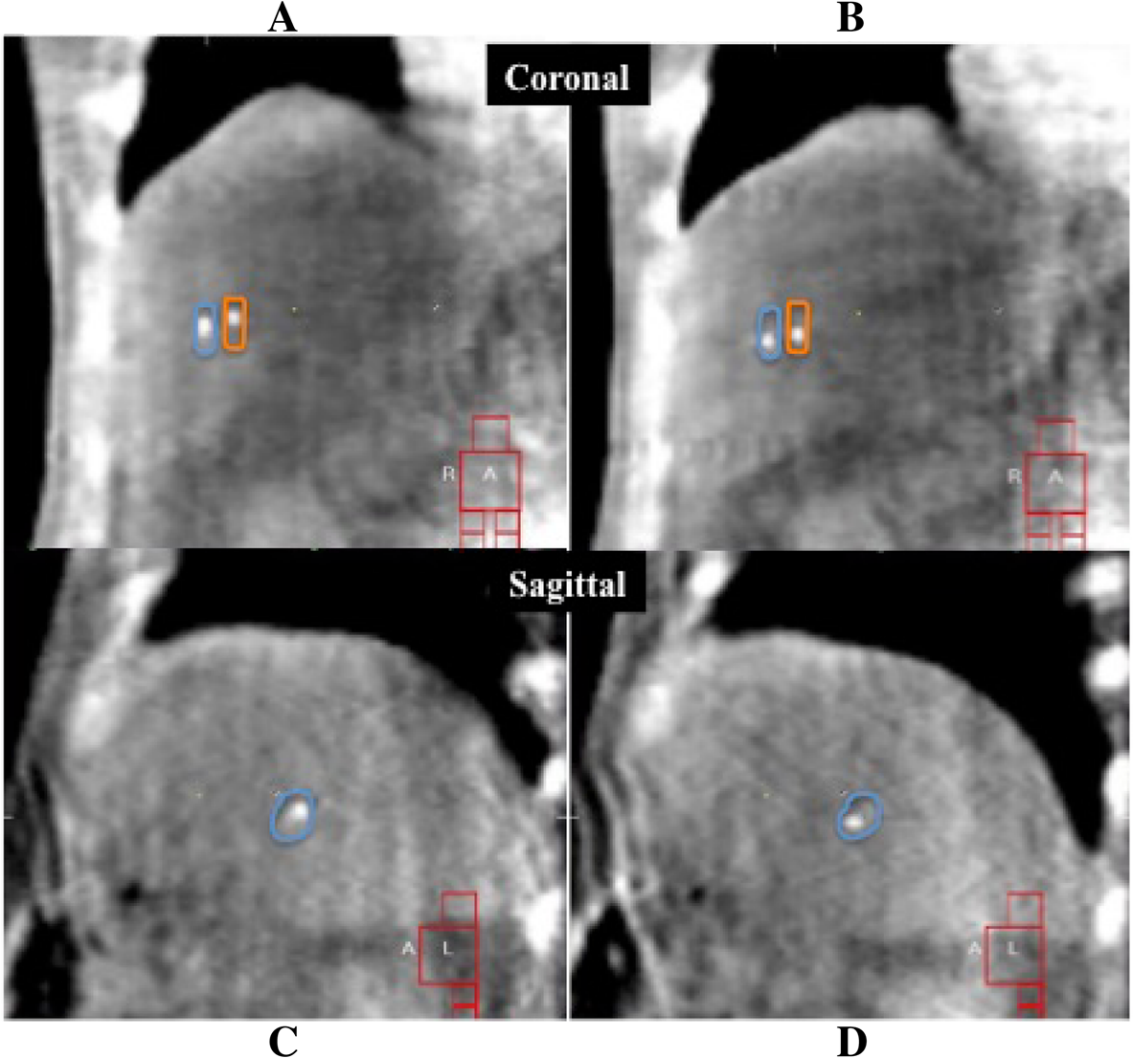
Four-dimensional cone-beam computed tomography (4D-CBCT) images of the end-exhalation **a**, **c** and end-inhalation **b**, **d**. The “internal marker target volume (IMTV)” structure (blue and orange) that reflected the motion of fiducial markers observed in 4D-CBCT were used for target localization in liver SBRT

On the same day of planning simulation CT, a 4D-CBCT scan was performed to evaluate liver tumor motion for the planning. The patient was positioned in the body immobilization system and aligned at the machine’s isocenter. The 4D-CBCT scans were acquired using the Elekta Symmetry System (Elekta Oncology Systems, Crawley, UK) and the projection data were sorted into 10 respiratory-phase bins [15]. The acquisition parameters were set in the small mode to 120 kV, 20 mA, 16 ms per frame, and 2-mm slice thickness with an acquisition time of 4 min. The small mode is designed to obtain projection data from a 200° gantry rotation with an FOV of 27 cm × 26 cm. The 4D-CBCT data were reconstructed using a 2-mm voxel size. Tumor motion was measured using Elekta XVI software (version 4.5; Elekta Oncology Systems, Crawley, UK). The translational distances at the center positions of the fiducial markers (COM) from all 10 phases on the 4D-CBCT images were measured as the extent of the liver tumor motion in the left-right (LR), anterior-posterior (AP), and superior-inferior (SI) directions (Fig. 1). The COM was defined as the maximum intensity position of the image pixels (minimum pixel size: 0.1 mm) using the image probe function of XVI software. The coordinates of COM were determined on the basis of each cross-sectional image, and the maximum distances of the coordinate were calculated as the extent of the motion of the liver tumor in LR, AP, and SI directions. In each patient, fiducial markers were measured individually, with an average of 1.8 markers, and a total of 18 markers [7].

### Treatment planning

GTV was defined by reference to the contrast-enhanced CT images and diagnostic magnetic resonance images. Clinical target volume (CTV) was defined with 3D margins of 0–3 mm to the GTV. The ITV was defined by applying margins to the CTV, which were based on the calculated results of the tumor motion. The planning target volume (PTV) was defined by adding margins ranging from 1–3 mm, 1–3 mm, and 3–6 mm to the ITV for the LR, AP, and SI directions, respectively. All structures of these tumor and target volumes, and the organs at risk (e.g., liver, gastrointestinal tract, spinal cord, and kidneys) were delineated on the slow CT images. Each fiducial marker was also delineated, and a covering volume with margins determined according to calculated results of the tumor motion was generated on the slow CT images, referred to as the “internal marker target volume” (IMTV) [8]. All slow CT data and structures were exported into the Elekta XVI software as the reference for image guidance.

Treatment planning was performed using 8–9 coplanar- and noncoplanar fields with a 6-MV and/or 10-MV photon beam on an Elekta Synergy with Agility multileaf collimator (Elekta Oncology Systems). The beam angles were chosen to minimize the treatment time while avoiding transmission of the beam path through the organs at risk.

### Treatment delivery

For liver SBRT target localization, 4D-CBCT was performed in a similar manner as the planning simulation. Manual registration between images of 4D-CBCT and slow CT was performed using the axial, coronal, and sagittal views until moving images of the fiducial marker of all 10 phases in the 4D-CBCT images were symmetrically positioned within the IMTV structure in the slow CT images. (Fig. 1). After the confirmation of the appropriate registration on the Elekta XVI software, the treatment couch was repositioned with translational correction, and liver SBRT was delivered. Immediately after treatment, a 4D-CBCT scan was performed to assess the intrafractional setup error of the target position. The treatment time, which was defined as the time from the beginning of the pre-SBRT 4D-CBCT to that of the post-SBRT 4D-CBCT, ranged from 11.1 to 25.7 min, with an average of 17.7 min.

### Liver tumor motion analysis

The liver tumor motion analyses were performed based on the coordinates of the COM from all 10 phases on the 4D-CBCT images using the image probe function of XVI software. For each patient, the mean and standard deviation (SD) of liver tumor motion, and inter- and intrafractional motion changes were calculated based on the 4D-CBCT images of the planning simulation, pre- and post-SBRT. The interfractional motion change was calculated as the difference in the liver tumor pre-SBRT relative to that of the planning simulation for each fraction. The intrafractional motion change was calculated as the difference between the liver tumor of pre- and post-SBRT for each fraction. Significant inter- and intrafractional changes in liver tumor motion were defined as a change of >3 mm, which corresponded to the threshold for the planning CT image resolution. Pearson correlation coefficients were calculated to evaluate correlations between liver tumor motion of the planning simulation and the mean liver tumor motion of the pre-SBRT. Statistical significance was defined as P < 0.05. All statistical calculations were performed with SPSS software, version 24.0 (SPSS Inc., Chicago, IL, USA).

## Results

### Correlation between liver tumor motion of the planning simulation and pre-SBRT

**Fig. 2:**
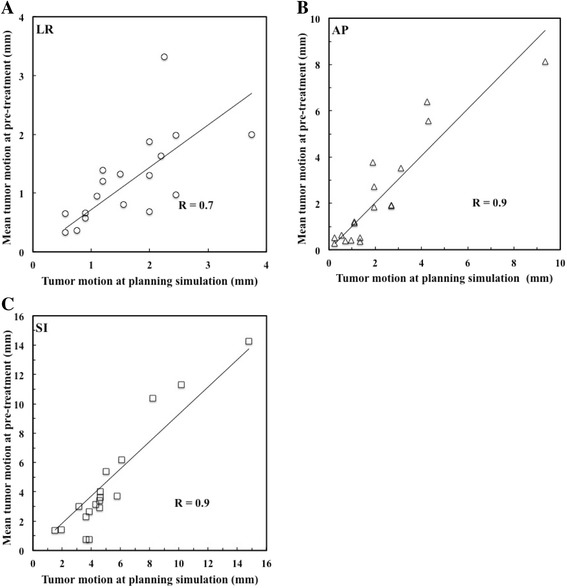
Correlation between liver tumor motion measured by planning simulation 4D-CBCT and mean liver tumor motion measured by pre-SBRT 4D-CBCT during five fraction treatments in the **a** left-right (LR), **b** anterior-posterior (AP), and **c** superior-inferior (SI) directions

For the 10 patients, the mean (± SD) liver tumor motions of the planning simulation were 1.7 ± 0.8 mm (range, 0.6–3.8 mm), 2.4 ± 2.2 mm (range, 0.3–9.4 mm), and 5.3 ± 3.3 mm (range, 1.5–14.8 mm) in the LR, AP, and SI directions, respectively. The mean (± SD) liver tumor motions of pre-SBRT were 1.2 ± 0.7 mm (range, 0.3–3.3 mm), 2.3 ± 2.3 mm (range, 0.3–8.1 mm), and 4.5 ± 3.8 mm (range, 0.7–14.3 mm) in the LR, AP, and SI directions, respectively. Figure 2 shows the correlation between the liver tumor motion of the planning simulation and the mean liver tumor motion of the pre-SBRT. There was a strong significant correlation in the LR (*R* = 0.7, *P* < 0.01), AP (*R* = 0.9, *P* < 0.01), and SI (*R* = 0.9, *P* < 0.01) directions.

### Inter- and intrafractional changes in liver tumor motion

**Fig. 3:**
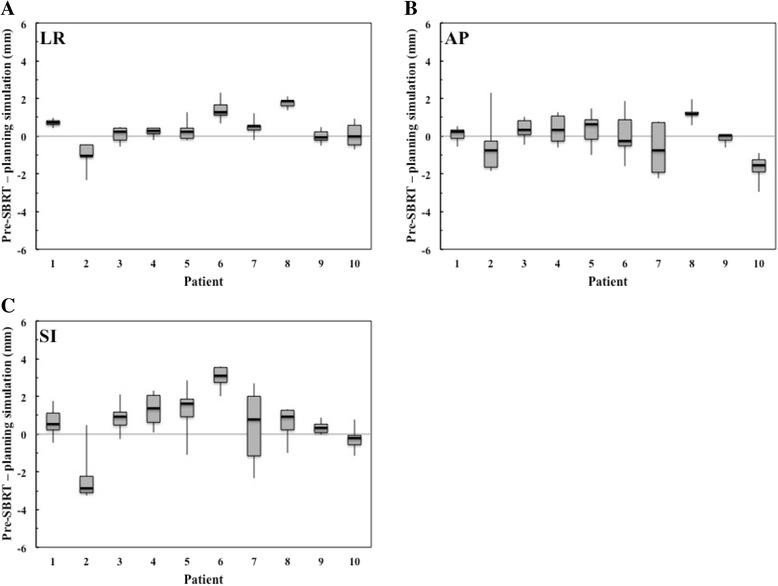
Box plots of interfractional changes in liver tumor motion (pre-SBRT - planning simulation) by patient in the **a** left-right (LR), **b** anterior-posterior (AP), and **c** superior-inferior (SI) directions. Lines represent ranges of liver tumor motion changes; boxes represent range from 25th to 75th percentile in liver tumor motion changes

**Fig. 4:**
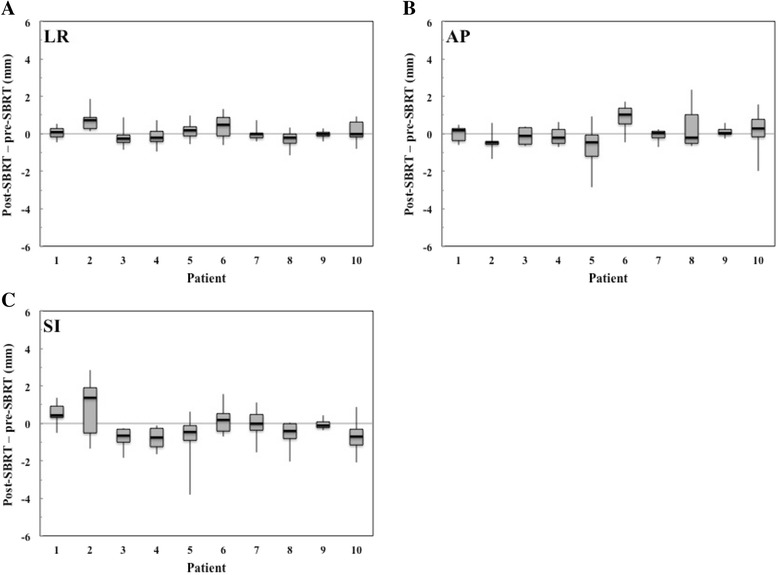
Box plots of intrafractional changes in liver tumor motion (post-SBRT - pre-SBRT) by patient in the **a** left-right (LR), **b** anterior-posterior (AP), and **c** superior-inferior (SI) directions. Lines represent ranges of liver tumor motion changes; boxes represent range from 25th to 75th percentile in liver tumor motion changes

The inter- and intrafractional liver tumor motion changes are displayed graphically in Figs. 3 and 4, respectively. Mean (± SD) absolute interfractional changes in the liver tumor motion were 0.6 ± 0.5 mm (range, 0.1–2.4 mm), 0.8 ± 0.7 mm (range, 0.1–3.0 mm), and 1.3 ± 1.0 mm (range, 0.1–3.6 mm) in the LR, AP, and SI directions, respectively. Interfractional liver tumor motion changes of >3 mm occurred in 10% of treatment fractions, in the SI direction alone. Mean (± SD) absolute intrafractional changes in liver tumor motion were 0.4 ± 0.3 mm (range, 0.1–1.9 mm), 0.6 ± 0.5 mm (range, 0.1–2.9 mm), and 0.7 ± 0.7 mm (range, 0.1–3.8 mm) in the LR, AP, and SI directions, respectively. Intrafractional liver tumor motion changes of >3 mm occurred in 2% of treatment fractions, in the SI direction alone.

## Discussion

The 4D-CBCT accurately represented the tumor motion range during reconstruction by sorting the projection data into multiple respiratory phases according to a respiration signal [15]. From the results of liver tumor motion, based on this state-of-the-art imaging modality, there was a strong correlation between liver tumor motion on the planning simulation and pre-SBRT. These data suggested that liver tumor motion at the planning simulation could represent liver tumor motion during SBRT. Therefore, 4D-CBCT can be introduced for not only treatment but also planning of liver SBRT as a useful modality for the evaluation of tumor motion in the institutions that do not have 4D-CT.

In the present study, the liver tumor motion pattern, based on 4D-CBCT, was approximately 2–3 times larger in the SI direction compared with the LR and AP directions. These motion patterns were similar to those in previous studies that utilized RTRT system, 4D-CT, and CBCT projection data [2, 10]. Kitamura et al. [2] investigated the liver tumor motion during free breathing without AC using RTRT and reported that the mean liver tumor motions were 4 ± 4 mm, 5 ± 3 mm, and 9 ± 5 mm in the LR, AP, and SI directions, respectively. Park et al. [10] investigated liver tumor motion in patients undergoing SBRT without AC using 4D-CT and CBCT, and found that liver tumor motions were 3.0 ± 2.0 mm, 5.1 ± 3.1 mm, and 17.9 ± 4 mm based on 4D-CT, and 2.8 ± 1.6 mm, 5.3 ± 3.1 mm, and 16.5 ± 5.7 mm based on CBCT, for the LR, AP, and SI directions, respectively. On the other hand, in the present study, the liver tumor motion ranges were smaller than those reported previously, most likely because we used AC [20]. Wunderink et al. [20] measured the effect of AC on the respiratory motion of liver tumors and concluded that AC reduced liver tumor motion to <5 mm in the all three directions in 10 of 12 patients. Therefore, AC use could effectively reduce 3D liver tumor motion.

The present study found that liver tumor motion changes were small in most patients who underwent liver SBRT with AC, and inter- and intrafractional motion changes of >3 mm were rare in any direction. Case et al. [4] investigated inter- and intrafractional liver motion changes in liver SBRT patients with or without AC, based on 4D-CBCT using the diaphragm position. They found that the mean absolute interfractional changes were 1.0 mm, 1.6 mm, and 1.7 mm and the mean intrafractional changes were 1.3 mm, 1.9 mm, and 1.6 mm for the LR, AP, and SI directions, respectively, which were larger than those observed in the present study. In addition to including the patients without AC, these differences likely arose because methods for evaluating liver tumor motion using the diaphragm position could result in overestimation due to motion-induced blurring of the diaphragm. For localization of the liver tumor, Wunderink et al. [9] and Zhang et al. [11] reported that the diaphragm position provided inaccurate SI set-up measurements and had an absolute error of >3 mm, compared with fiducial markers. Precise liver SBRT should be realized by the combined use of 4D-CBCT and fiducial markers at both the planning and treatment stages. Moreover, our results suggested that adding 3D margins of 3 mm or less to liver tumor motion at the planning simulation might be adequate to cover the tumor motion at the treatment if AC is available.

This present study had some limitations, including the relatively small number of patients. We evaluated intrafractional changes based on 4D-CBCT scans before and after liver SBRT because we could not measure intrafractional changes by monitoring the fiducial markers during treatment. The development of a system for performing in-treatment 4D-CBCT could address this issue [21].

## Conclusions

In this paper, we evaluated the tumor motion of liver SBRT with AC throughout the course of treatment based on 4D-CBCT and fiducial markers. Liver tumor motion at the planning simulation could represent liver tumor motion in patients undergoing SBRT. Significant inter- and intrafractional motion changes were rare in most patients who underwent liver SBRT with AC. Precise liver SBRT should be realized by the combined use of 4D-CBCT and fiducial markers at both the planning and treatment stages.

## Abbreviations

AC: Abdominal compression
AP: Anterior-posterior
CBCT: Cone-beam computed tomography
COM: Center positions of the fiducial markers
CT: Computed tomography
CTV: Clinical target volume
FOV: Field of view
GTV: Gross tumor volume
IMTV: Internal marker target volume
ITV: Internal target volume
LR: Left-right
PTV: Planning target volume
RTRT: Real-time tumor tracking radiotherapy
SBRT: Stereotactic body radiation therapy
SD: Standard deviation
SI: Superior-inferior

## References

[1] Wulf J, Guckenberger M, Haedinger U, Oppitz U, Mueller G, Baier K (2006). Stereotactic radiotherapy of primary liver cancer and hepatic metastases. Acta Oncol.

[2] Kitamura K, Shirato H, Seppenwoolde Y, Shimizu T, Kodama Y, Endo H (2003). Tumor location, cirrhosis, and surgical history contribute to tumor movement in the liver, as measured during stereotactic irradiation using a real-time tumor-tracking radiotherapy. Int J Radiat Oncol Biol Phys.

[3] Guckenberger M, Sweeney RA, Wilbert J, Krieger T, Richter A, Baier K (2008). Image-guided radiotherapy for liver cancer using respiratory correlated computed tomography and cone-beam computed tomography. Int J Radiat Oncol Biol Phys.

[4] Case RB, Moseley DJ, Sonke JJ, Eccles CL, Dinniwell RE, Kim J (2010). Interfraction and intrafraction changes in amplitude of breathing motion in stereotactic liver radiotherapy. Int J Radiat Oncol Biol Phys.

[5] Abbas H, Chang B, Chen ZJ (2014). Motion management in gastrointestinal cancers. J Gastrointest Oncol.

[6] Beddar AS, Briere TM, Balter P, Pan T, Tolani N, Ng C (2008). 4D-CT imaging with synchronized intravenous contrast injection to improve delineation of liver tumors for treatment planning. Radiother Oncol.

[7] Rankine L, Wan H, Parikh P, Maughan N, Poulsen P, DeWees T (2016). Cone-beam computed tomography internal motion tracking should be used to validate 4-dimensional computed tomography for abdominal radiation therapy patients. Int J Radiat Oncol Biol Phys.

[8] Heinz C, Gerum S, Freislederer P, Ganswindt U, Roeder F, Corradini S (2016). Feasibility study on image guided patient positioning for stereotactic body radiation therapy of liver malignancies guided by liver motion. Radiat Oncol.

[9] Wunderink W, Méndez Romero A, Seppenwoolde Y, de Boer H, Levendag P, Heijmen B (2010). Potentials and limitations of guiding liver stereotactic body radiation therapy set-up on liver-implanted fiducial markers. Int J Radiat Oncol Biol Phys.

[10] Park JC, Park SH, Kim JH, Yoon SM, Song SY, Liu Z (2012). Liver motion during cone beam computed tomography guided stereotactic body radiation therapy. Med Phys.

[11] Zhang T, Wang W, Jin J (2013). Study of match methods in cone beam computed tomography online correction for postoperative radiotherapy in liver cancer patients. Chin J Radiat Oncol.

[12] Keall PJ, Mageras GS, Balter JM, Emery RS, Forster KM, Jiang SB (2006). The management of respiratory motion in radiation oncology report of AAPM Task Group 76. Med Phys.

[13] Rietzel E, Chen GT, Choi NC, Willet CG (2005). Four-dimensional image-based treatment planning: target volume segmentation and dose calculation in the presence of respiratory motion. Int J Radiat Oncol Biol Phys.

[14] Xi M, Liu MZ, Deng XW, Zhang L, Huang XY, Liu H (2007). Defining internal target volume (ITV) for hepatocellular carcinoma using four-dimensional CT. Radiother Oncol.

[15] Sonke JJ, Zijp L, Remeijer P, van Herk M (2005). Respiratory correlated cone beam CT. Med Phys.

[16] Sweeney RA, Seubert B, Stark S, Homann V, Müller G, Flentje M (2012). Accuracy and inter-observer variability of 3D versus 4D cone-beam CT based image-guidance in SBRT for lung tumors. Radiat Oncol.

[17] Nakagawa K, Haga A, Kida S, Masutani Y, Yamashita H, Takahashi W (2013). 4D registration and 4D verification of lung tumor position for stereotactic volumetric modulated arc therapy using respiratory-correlated cone-beam CT. J Radiat Res.

[18] Park JC, Park SH, Kim JH, Yoon SM, Kim SS, Kim JS (2011). Four-dimensional cone-beam computed tomography and digital tomosynthesis reconstructions using respiratory signals extracted from transcutaneously inserted metal markers for liver SBRT. Med Phys.

[19] Shirato H, Harada T, Harabayashi T, Hida K, Endo H, Kitamura K (2003). Feasibility of insertion/implantation of 2.0-mm-diameter gold internal fiducial markers for precise setup and real-time tumor tracking in radiotherapy. Int J Radiat Oncol Biol Phys.

[20] Wunderink W, Romero AM, de Kruijf W, de Boer H, Levendag P, Heijmen B (2008). Reduction of respiratory liver tumor motion by abdominal compression in stereotactic body frame, analyzed by tracking fiducial markers implanted in liver. J Radiat Oncol Biol Phys.

[21] Takahashi W, Yamashita H, Kida S, Masutani Y, Sakumi A, Ohtomo K (2013). Verification of planning target volume settings in volumetric modulated arc therapy for stereotactic body radiation therapy by using in-treatment 4-dimensional cone beam computed tomography. Int J Radiat Oncol Biol Phys.

